# Effects of Constant and Doublet Frequency Electrical Stimulation Patterns on Force Production of Knee Extensor Muscles

**DOI:** 10.1371/journal.pone.0155429

**Published:** 2016-05-11

**Authors:** Carole Cometti, Nicolas Babault, Gaëlle Deley

**Affiliations:** 1 INSERM U1093, "Cognition, Action, et Plasticité Sensorimotrice", Faculté des Sciences du Sport, Université de Bourgogne-Franche-Comté, Dijon, France; 2 Centre d'Expertise de la Performance, Faculté des Sciences du Sport, Université de Bourgogne-Franche-Comté, Dijon, France; Shanghai Jiao Tong University, CHINA

## Abstract

This study compared knee extensors’ neuromuscular fatigue in response to two 30-minute stimulation patterns: constant frequency train (CFT) and doublet frequency train (DFT). Fifteen men underwent two separate sessions corresponding to each pattern. Measurements included torque evoked by each contraction and maximal voluntary contractions (MVC) measured before and immediately after the stimulation sessions. In addition, activation level and torque evoked during doublets (Pd) and tetanic contractions at 80-Hz (P80) and 20-Hz (P20) were determined in six subjects. Results indicated greater mean torque during the DFT stimulation session as compared with CFT. But, no difference was obtained between the two stimulation patterns for MVC and evoked torque decreases. Measurements conducted in the subgroup depicted a significant reduction of Pd, P20 and P80. Statistical analyses also revealed bigger P20 immediate reductions after CFT than after DFT. We concluded that DFT could be a useful stimulation pattern to produce and maintain greater force with quite similar fatigue than CFT.

## Introduction

Functional Electrical Stimulation (FES) is a technique permitting to evoke muscle contractions via the application of electrical pulses. This method can produce functionally useful movements such as leg flexion/extension, standing, walking, cycling, and even rowing [1–4]. It is therefore a useful way to produce force and restore movements to perform daily activities for people with spinal cord injury [5,6]. However, in spite of its utility [7,8], rapid fatigue associated with electrical stimulation limits the effectiveness of FES. Indeed, the lack of activation due to neurological damages after a spinal cord injury renders skeletal muscle highly susceptible to fatigue. Moreover, FES recruits motor units in a nonselective, spatially fixed, with temporally synchronous pattern [9]. As a consequence, in rehabilitation, the ideal stimulation pattern would be one that produces sufficiently high forces while minimizing fatigue. To that purpose, different stimulation trains have been proposed and compared in the literature [10,11].

FES traditionally consists of constant-frequency trains (CFT). Briefly, it involves tetanic stimulations separated by regular interpulse intervals. However, it has been shown that variable frequency trains may limit fatigue development and is more efficient at generating force in fresh and fatigued muscles as compared to CFT [10,12–15]. Variable frequency trains generally begins with two or three high-frequency stimuli (called doublet or triplet, respectively) followed by regularly spaced single pulses with longer intervals [15,16]. Another form of variable frequency trains, called doublet frequency trains (DFT), differs from the preceding variable frequency train pattern since doublets are repeated during the whole contraction and not only at the onset of stimulation trains. It is therefore composed of consecutive high-frequency doublets separated by longer interdoublet intervals [10,11]. Doublets are used since they have been shown to be preferable than single pulses, triplets or more for force improvements and fatigue reduction [17]. DFT pattern, like other variable frequency trains, is based on the nonlinear force summation [17] and on the catchlike muscle property [16]. This pattern has been shown to limit fatigue due to increased calcium release from the sarcoplasmic reticulum and increased stiffness [10,11]. It would therefore reduce low-frequency muscle fatigue.

Although some authors have shown that short duration (generally < 1 s) DFT could be of interest to produce high forces and reduce muscle fatigue [10,11], little is known when using stimulation durations longer than 1 s. Because time under tension is an important determinant for long-term training adaptations [18], long durations are mandatory for FES rehabilitation and strengthening procedures in patients. Therefore, the aim of this study was to investigate whether DFT stimulation pattern was better than CFT for force production during a single session and fatigue reduction after the session. Able-bodied participants were considered in this preliminary study. In addition, because little is known regarding DFT fatigue mechanisms, the secondary aim of the present study was to determine neuromuscular fatigue origins after the stimulation session in a small subgroup thanks to mechanical properties, activation level and low-frequency fatigue assessments.

## Methods

### Participants and Ethics Statements

Fifteen physically active men, with no previous history of injury to the lower limbs and familiar with electrical stimulation, participated in this study (age: 30 ± 5 yrs, body mass: 73.9 ± 9.1 kg, height: 176.7 ± 7.5 cm). Subjects were all instructed to refrain from strenuous activity at least 24 hours before all testing sessions. The study was conducted according to the declaration of Helsinki and approval for the project was obtained from the Sport Science Faculty review board of the Burgundy University. Each participant read and signed a written informed consent document outlining the procedures, objectives and potential risks of the experiment.

### Experimental Design

**Fig 1:**
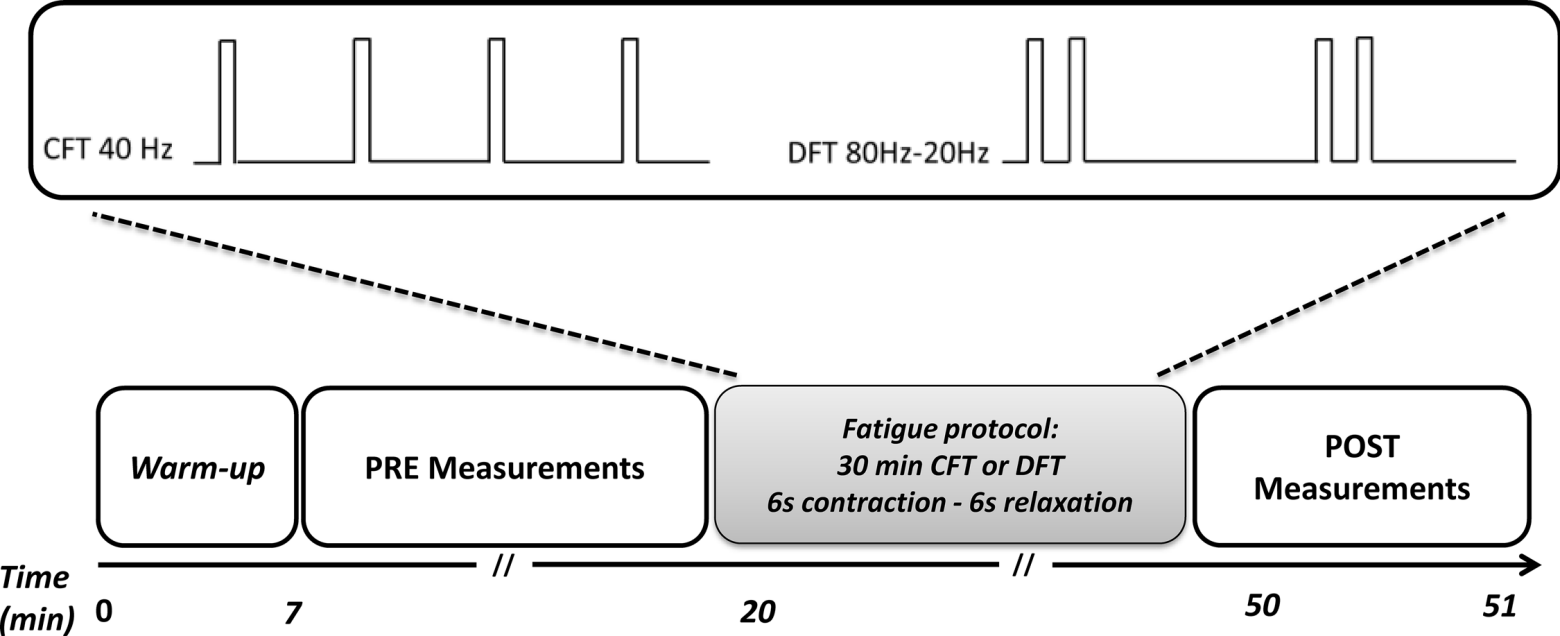
Stimulation pattern and schematic view of the experimental protocol. Upper panel presents the two stimulation patterns. Lower panel presents the experimental protocol. Warm-up consisted of 10 progressive voluntary contractions until the maximal voluntary contraction (MVC). The experimental session then began with different measurements (*PRE measurement*s) including the determination of the optimal stimulation intensity for doublets, tetanic contractions and the session's pattern and tests with two MVCs and two 500 ms tetanic contractions at 20 and 80 Hz. Then, subjects underwent 30 minutes with one stimulation pattern. After the session, *POST measurements* were performed including two MVCs, doublets, two 500 ms tetanic contractions at 20 and 80 Hz.

One week before the protocol onset, subjects performed a familiarization session for electrical stimulation procedures and tests. Afterwards, all underwent two testing sessions separated by at least 72 hours. Sessions were randomly assigned between the two stimulation patterns: CFT and DFT. Tests were conducted before (PRE) and immediately after (POST) each 30-minute stimulation procedures to investigate neuromuscular fatigue. Additional neuromuscular measurements were performed in six subjects in order to investigate fatigue origins. The global experimental design is shown in Fig 1.

During the whole experimental procedure, subjects were seated on an isokinetic dynamometer (Quick-set Biodex system 4, Biodex corporation, Shirley, NY). Velcro straps were attached across the trunk for stability. The dominant leg was fixed to the dynamometer lever-arm. The axis of rotation of the dynamometer was aligned to the lateral femoral condyle that indicates the anatomical joint axis of the knee. All measurements were conducted at a 90° knee joint angle (0° corresponding to full extension) on relaxed quadriceps. Stimulation was performed with a Compex 2 stimulator (Compex, Medicompex SA, Ecublens, Switzerland) using two 10 × 5 cm adhesive electrodes. One positive electrode was placed distally close to vastus lateralis and vastus medialis motor points. The negative electrode was placed proximally 5–7 cm below the inguinal ligament.

Following set-up, each session began with a standardized warm-up. It was composed of ten progressive voluntary isometric contractions until the maximal voluntary contraction (MVC). Then, two 5-s MVC were performed separated by 15 s. Subjects were strongly encouraged, and the best result was retained for analyses. This value was also used to determine the sessions' stimulation intensities (see details below).

Stimulation patterns were then applied in a random order. According to stimulation characteristics, CFT and DFT were matched with pulse number. The CFT pattern, previously used [15], consisted of 6 s contractions (no ramp) generated by a continuous 40 Hz stimulation with a rectangular pulse of 450 μs. DFT pattern consisted of 6 s contractions (no ramp) generated by successive 80 Hz doublets delivered at 20 Hz with a rectangular pulse of 450 μs. Because 6 s contractions have been found to be effective for strength increases with electrical stimulation programs [19], each contraction was 6 s long followed by a 6-s period of rest. The stimulation intensity was first determined using successive increasing contractions until evoked torque was equal to 30% of the MVC. The determination of stimulation intensity was conducted using a maximum of five increasing intensities. After 10 minutes rest, stimulation patterns were applied for a total duration of 30 minutes. This total duration was previously used during FES programs and for studies investigating different stimulation pattern [3,15]. Immediately after these stimulation procedures, two 5-s MVC were repeated with 15-s rest for POST measurements.

### Additional Measurements

A subgroup composed of six subjects underwent additional measurements in order to investigate the origins of neuromuscular fatigue. To that purpose, electrical impulses were delivered through the same surface electrodes than those used for CFT and DFT procedures. Square-wave pulses were produced by an electrical stimulator (Digitimer DS7, Hertfordshire, England). Optimal stimulation intensity was determined isometrically using single twitches (1-ms duration, 400 V maximum voltage and intensity ranging from 60 to 200 mA) separated by 5 s, with progressively increasing intensity until twitch torque failed to increase. Once the maximal intensity was found, it was further increased by 25%. This supramaximal intensity was used during the remainder of the session with doublet stimulations (two electrical impulses separated by 10 ms; 100 Hz as previously used [20]).

Doublets were applied (i) at rest, before each MVC, to assess contractile properties, (ii) during the MVC plateau (superimposed doublet) and (iii) 1 s after the MVC (control doublet). In addition, two 500-ms tetanic trains were delivered at 80 Hz (P80) and then 20 Hz (P20) frequencies according to the methodology described by Martin et al. [20]. Thirty seconds rest was used between each train. Doublets and tetanic trains were delivered before and after the 30-min stimulation procedure. The purpose was to investigate fatigue origins and to discriminate high and low-frequency fatigue; a predominant torque decrease following high-frequency stimulations (P80) or low-frequency stimulations (P20), respectively [21].

### Data Analyses

Mechanical traces were digitized online at a 1,000 Hz sampling frequency and stored for analyses (Biopac MP150 A/D converter and AcqKnowledge v4.2 software, Biopac Systems Inc., Santa Barbara, CA). Fatigue was quantified after the stimulation sessions using (i) PRE and POST MVC and (ii) by using the torque produced during the first and last five evoked contractions of the 30-minute stimulation procedure. Torque produced during the entire stimulation sessions was also averaged and termed 'mean torque'.

For additional measurements, peak torque developed during each doublet (Pd) and P20 and P80 trains were measured. Superimposed and control doublets were used to calculate the activation level using the following formula: $Activation\;level=\;\left(1-\frac{superimposed\;doublet}{control\;doublet}\right)\times 100$ [22]. Lastly, the P20-to-P80 ratio (P20/P80) was calculated as an index of low-frequency fatigue.

### Statistical Analyses

Data are expressed as mean values ± SD. A two-way analysis of variance (ANOVA) with repeated-measures (pattern × time) was used. Pattern refers to CFT vs. DFT, and time to PRE vs. POST. When main effects or interactions were observed, post hoc Newman-Keuls tests were used. In addition, a paired t-test was used to compare mean torque and percentage decreases obtained with the two stimulation patterns. Values obtained from additional measurements were compared using a non-parametric test (Wilcoxon). Effect sizes (d) were also determined, with values of 0.2, 0.5, 0.8 and above 1.2 considered to represent small, medium, large and very large differences, respectively [23]. Statistical significance was accepted with P<0.05.

## Results

**Fig 2:**
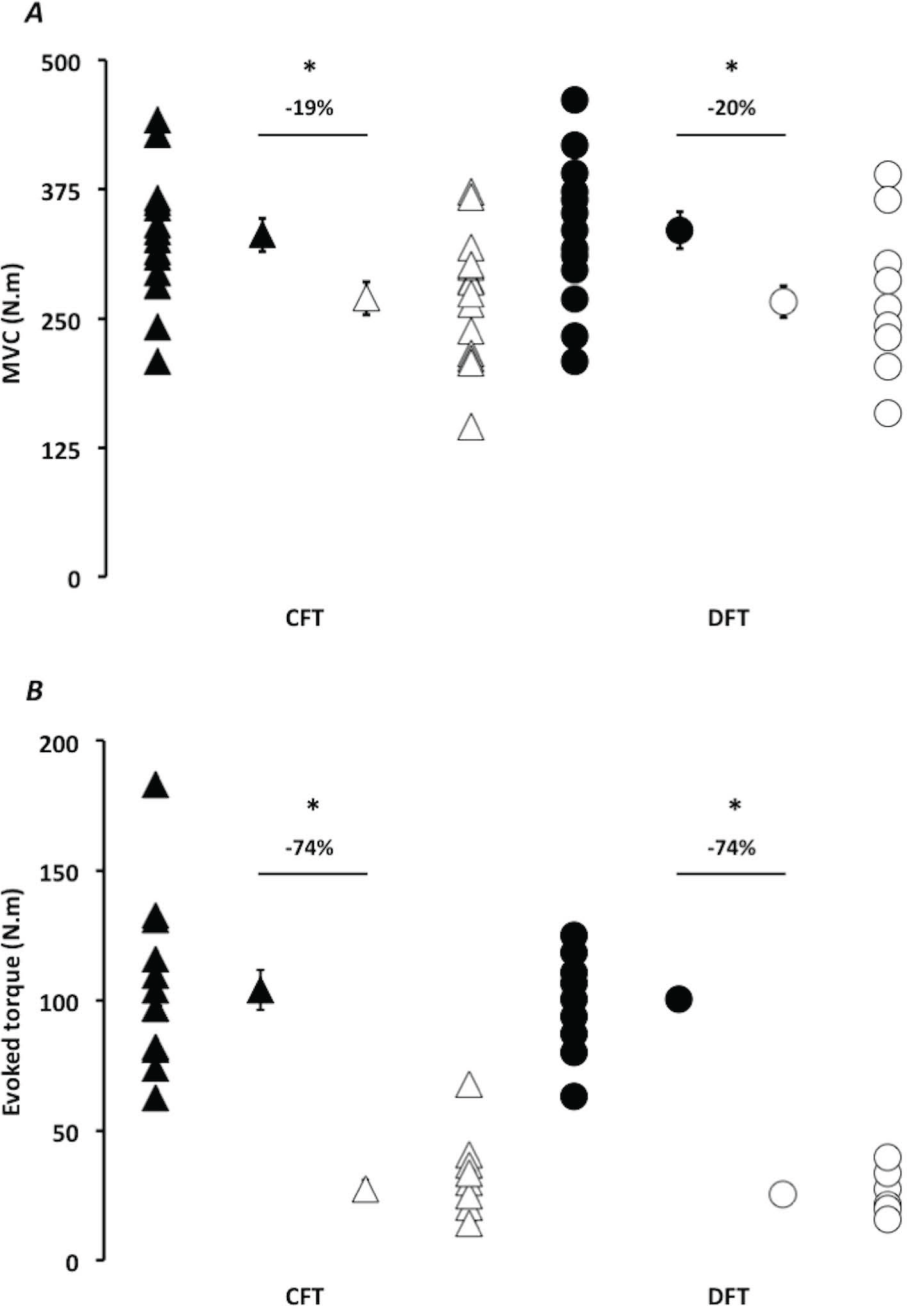
Voluntary and evoked torque in pre- and post-fatigue conditions. (A) Maximal voluntary torque (Nm) values obtained before (black symbols) and immediately after (white symbols) the constant-frequency trains program (CFT) and doublet frequency train (DFT). (B) Average torque evoked during the first 5 (black symbols) and the last 5 contractions (white symbols) of each stimulation program. Values in the inner part of CFT and DFT represented mean values ± SD Values in the outer part of CFT and DFT represented individual values. *: statistical differences between PRE and POST values (*P*<0.05).

The present study revealed a significant difference (P<0.05) between CFT and DFT in mean torque produced during the entire stimulation procedure (28.6 ± 8.6 N.m and 33.7 ± 7.8 N.m, respectively; d = 2.17, very large difference). No stimulation pattern effect was shown for PRE and POST MVC and the first five and last five contractions of the stimulation procedures. A significant main time effect was only observed (P<0.05; Fig 2). PRE and POST MVC decreased from 333.4 ± 62.3 N.m to 267.8 ± 58.2 N.m (average between CFT and DFT, d = 1.08, large difference). Evoked torque decreased from 102.4 ± 22.7 N.m during the first five contractions to 26.4 ± 11.4 N.m during the last five contractions (average between CFT and DFT, d = 4.23, very large difference).

Considering the subgroup, a significant decrease (P<0.05) in Pd, P20 and P80 was measured between PRE and POST values in both the CFT and DFT conditions (Table 1). Although small alterations appear, no significant differences were obtained for the other parameters (activation level and P20/P80). When comparing both the CFT and DFT conditions, statistical analyses only revealed differences in P20 changes. P20 alteration was significantly greater (P<0.05) after CFT than DFT (-64.9 ± 9.4% and -48.0 ± 11.9%, respectively, d = 1.57, very large difference). No other difference was obtained.

**Table 1:** Neuromuscular parameters registered in the subgroup before (PRE) and after (POST) stimulation procedures.

	Stimulation pattern	PRE	POST	Effect size (d)
Pd (N.m)	CFT*	71.7 ± 7.0	58.2 ± 7.3	1.89, very large
	DFT*	72.6 ± 9.5	60.8 ± 6.9	1.69, very large
Activation level (%)	CFT	95.2 ± 4.3	93.9 ± 4.8	0.28, small
	DFT	97.6 ± 2.3	86.8 ± 15.6	0.86, large
P20 (N.m)	CFT*	102.8 ± 26.2	38.3 ± 19.1	2.81, very large
	DFT*	120.4 ± 32.7	66.0 ± 30.5	1.72, very large
P80 (N.m)	CFT*	173.3 ± 52.7	80.7 ± 23.1	2.27, very large
	DFT*	171.1 ± 54.9	117.7 ± 41.9	1.09, large
P20/P80 ratio	CFT	0.61 ± 0.09	0.46 ± 0.17	1.11, large
	DFT	0.76 ± 0.31	0.57 ± 0.22	0.70, medium

CFT: constant frequency train; DFT: doublet frequency train, Pd: peak doublet, P20 and P80: torque produced during 20 Hz and 80 Hz tetanical stimulations.

*: significant difference between PRE and POST (P<0.05).

## Discussion

The aim of the present study was to compare force and fatigue during and after the application of two different stimulation patterns on knee extensor muscles. As expected, DFT appeared more effective for force production during a fatiguing procedure than CFT. However, fatigue was almost similar with both stimulation patterns. Measurements conducted in a subgroup revealed a difference with regards to low-frequency fatigue.

The main result of the present study was that, force produced during the stimulation session was greater with DFT than CFT. Such finding partly confirms previous experiments [10,12–15]. As compared with CFT, variable frequency trains appeared more efficient to increase force production during a single session. More specifically, the present study was the first to demonstrate that long durations' DFT (>1 s) could be a potentially useful stimulation pattern for rehabilitation procedures. For example, Scott et al. [24] only used a six-pulses DFT pattern with frequencies ranging from 10 to 100 Hz (i.e., <300 ms). Another study, conducted in isotonic conditions, applied DFT shorter than 800 ms to reach a target position [11]. However, this greater mean torque with short or long contraction duration, as compared to CFT, produced a similar amount of fatigue at the end of the session (similar evoked peak torque and MVC decreases).

Additional data, obtained in a six subjects subgroup, permitted to give more lights regarding fatigue origins. The lack of activation changes pointed out the primary role of peripheral fatigue rather than central. More specifically, although no significant differences were obtained for P20/P80 ratio, some alterations in low-frequency fatigue were noticed. The only difference obtained was that P20 decreased more with CFT as compared with DFT. This finding provides evidences that demonstrate a greater low-frequency fatigue with CFT than with DFT. Briefly, low-frequency fatigue is mostly due to Ca^2+^ alterations [21,25], while high-frequency fatigue is mostly attributed to extracellular ion concentrations [21,26]. This reduced low-frequency fatigue, observed here, has previously been suggested with variable frequency trains such as the DFT pattern [10,11,24]. Indeed, the rationale for using variable frequency trains relates to the fact that doublets have been shown to enhance calcium release from the sarcoplasmic reticulum and calcium sensitivity [27,28]. Therefore, these stimulation patterns are less susceptible to impairments in excitation-contraction coupling associated with low-frequency fatigue [29]. Moreover, DFT used here was associated with lower stimulation frequency than CFT. Beyond the effect of variable frequency trains, frequency may also influence such low- or high-frequency fatigue. Indeed, authors have shown that fatigue with low-frequency stimulation (10 Hz) was primarily liked to metabolic changes [30]. In contrast, fatigue with high-frequency stimulation was attributed to electrical activity failure (action potential propagation).

According to the reduced low-frequency fatigue during DFT, previous experiments suggested that this stimulation pattern was effective in both non-fatigued and fatigued conditions [11,24,31]. Our results were in apparent disagreement with these previous data. Indeed, torque produced during the last contractions of both the CFT and DFT sessions were similar. This discrepancy with the existing literature could be attributable to the different fatiguing durations. While 30 minutes were used here, the other studies induced fatigue with procedures around two minutes. Thus, doublets might not be efficient enough to overcome impairments of excitation-contraction coupling that results from reduced Ca^2+^ release from the sarcoplasmic reticulum in extremely fatiguing conditions. It means that differences between DFT and CFT would be more pronounced at the beginning of our stimulation procedure or with shorter stimulation sessions, i.e., with small levels of fatigue. Therefore, a study considering fatigue time course might give additional information. Nevertheless, taken as a whole, our results suggest that DFT was efficient to maintain a given force level longer, i.e., with a relatively low fatigue.

Variable frequency trains such as DFT are of interest because they are based on the nonlinear force summation [17,28] and on the muscle catchlike property [16]. Mechanisms have previously been described [12] and include previously discussed calcic processes. Other mechanisms are involved. Changes in muscle stiffness are now well documented. Indeed, doublet impulses are thought to take up the slack of series elastic components. They would increase stiffness and therefore would generate and transmit force more efficiently [32,33]. Multiple doublets during DFT resulted in repeated stiffness increases and thus better force production [11].

Another hypothesis, recently proposed [15], is related to motor unit recruitment in relation to stimulation characteristics such as frequency. Indeed, it is well known that electrical stimulation imposes continuous contractile activity to the same population of superficial muscle fibres with synchronous motor unit recruitment [34]. This almost constant recruitment will accelerate fatigue as compared to voluntary contractions [35,36]. Bickel et al. [37] suggested that the greater fatigue observed during electrical stimulation, as compared to voluntary contractions, is associated with the inability to alter recruitment pattern and/or inability to modulate firing frequency. Changing stimulation characteristics (pulse frequency, width and amplitude) have been proposed to be an essential strategy to mimic natural motor unit recruitment (recruitment of additional motor units when fibred initially recruited become fatigued) and therefore delay muscle fatigue [36]. Among the stimulation characteristics, frequency seems to have more effects on fatigue than pulse duration or amplitude [38]. These authors attributed their results to a decreased evoked torque relative to the activated muscle area. Others also shown that lowering stimulation frequencies could reduce fatigue development [39]. Whatever the mechanism, the lower stimulation frequency used between doublets for DFT, would therefore be of great interest to slow down muscle fatigue. Additional measurements are necessary to determine fatigue origins associated with these two stimulation patterns. For example, electrode positioning such as multiple electrodes over a muscle or direct nerve stimulation, has been shown to have a large influence in force production and fatigue time course [37].

In conclusion, the present study demonstrated that, even with long and numerous contractions, DFT could be a useful stimulation pattern to maintain high forces without any additional fatigue as compared to CFT. Mechanisms related to muscles' catchlike property such as increased stiffness, enhanced calcic processes and motor unit recruitment are expected. This stimulation strategy would increase the total force output during a given exercise session that could be of interest more particularly for FES rehabilitation procedures. Nevertheless, this preliminary study was conducted on able-bodied individuals and the efficiency of such stimulation procedure should be investigated in patients such as spinal cord injured and in combination with other stimulation pattern for training optimization [40]. Indeed, able-bodied and those with spinal cord injury widely differed in muscle composition and function and cardiovascular function, for example [6]. Also, the absence of sensation in spinal cord injured individuals might influence the effect of the different stimulation patterns. Finally, since the present study considered fatigue at the end of the procedure, it could be of interest to replicate this study to investigate fatigue time course with shorter stimulation sessions producing less fatigue.
